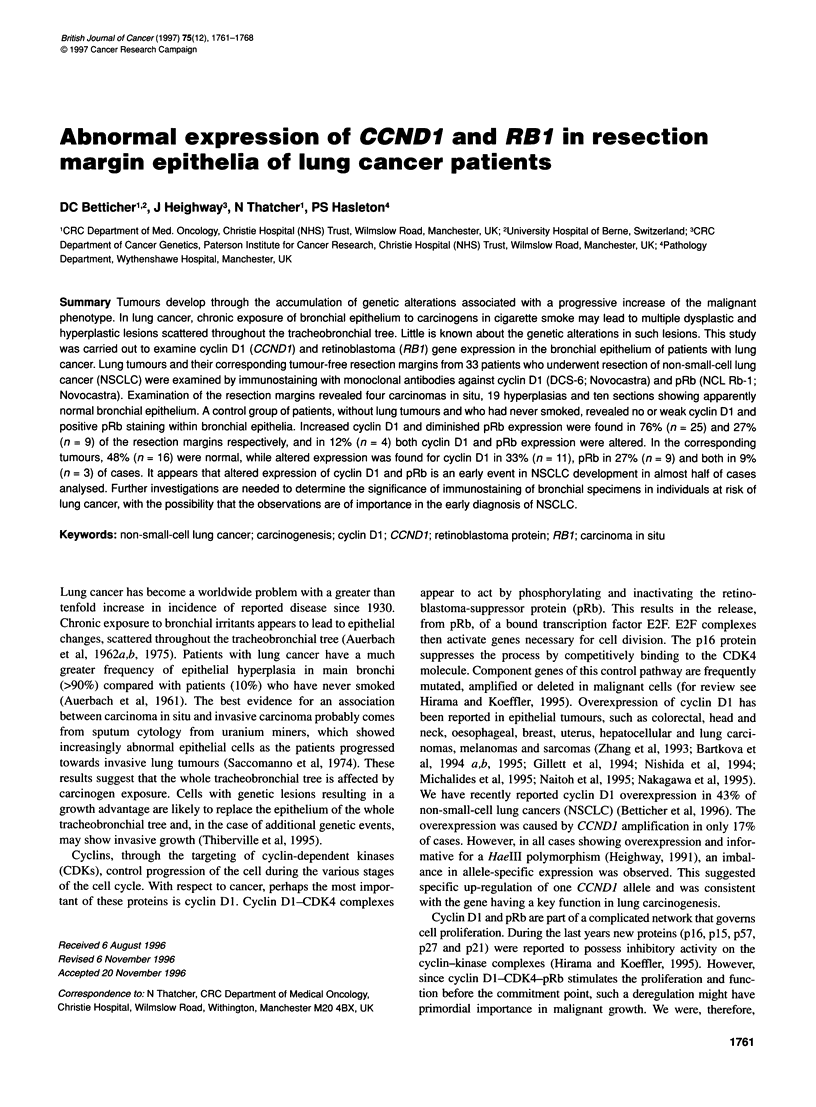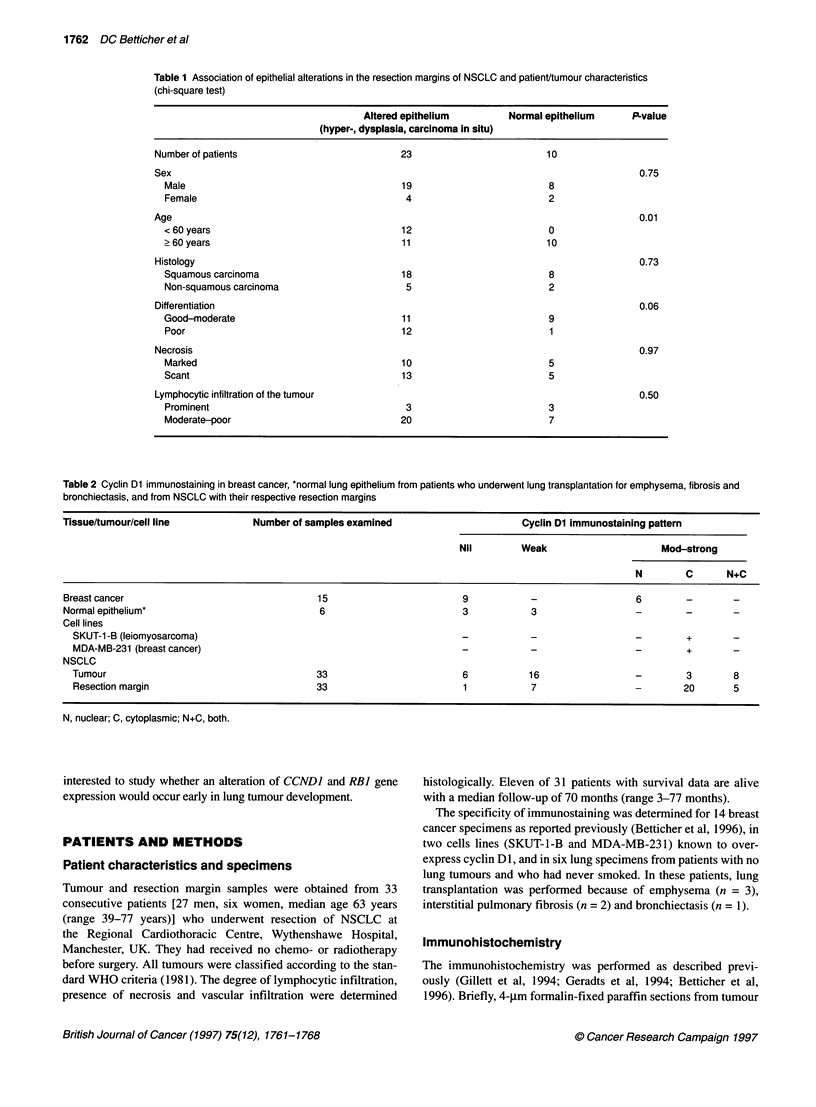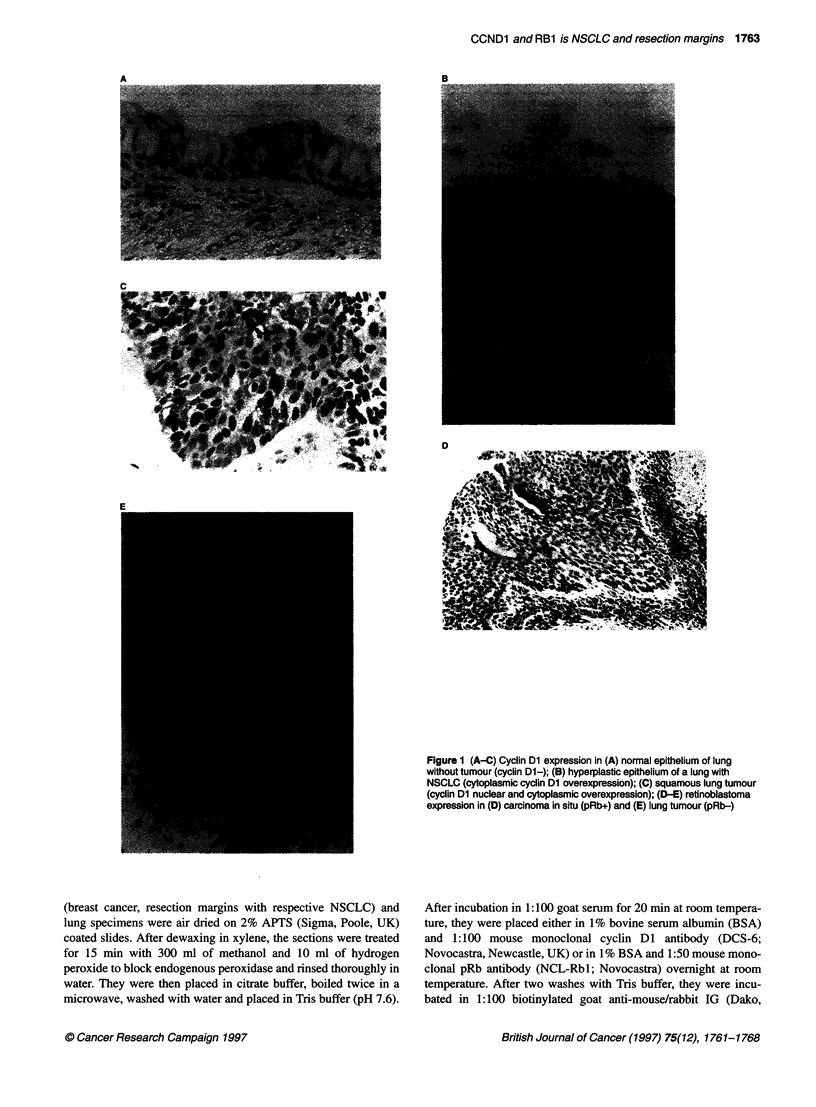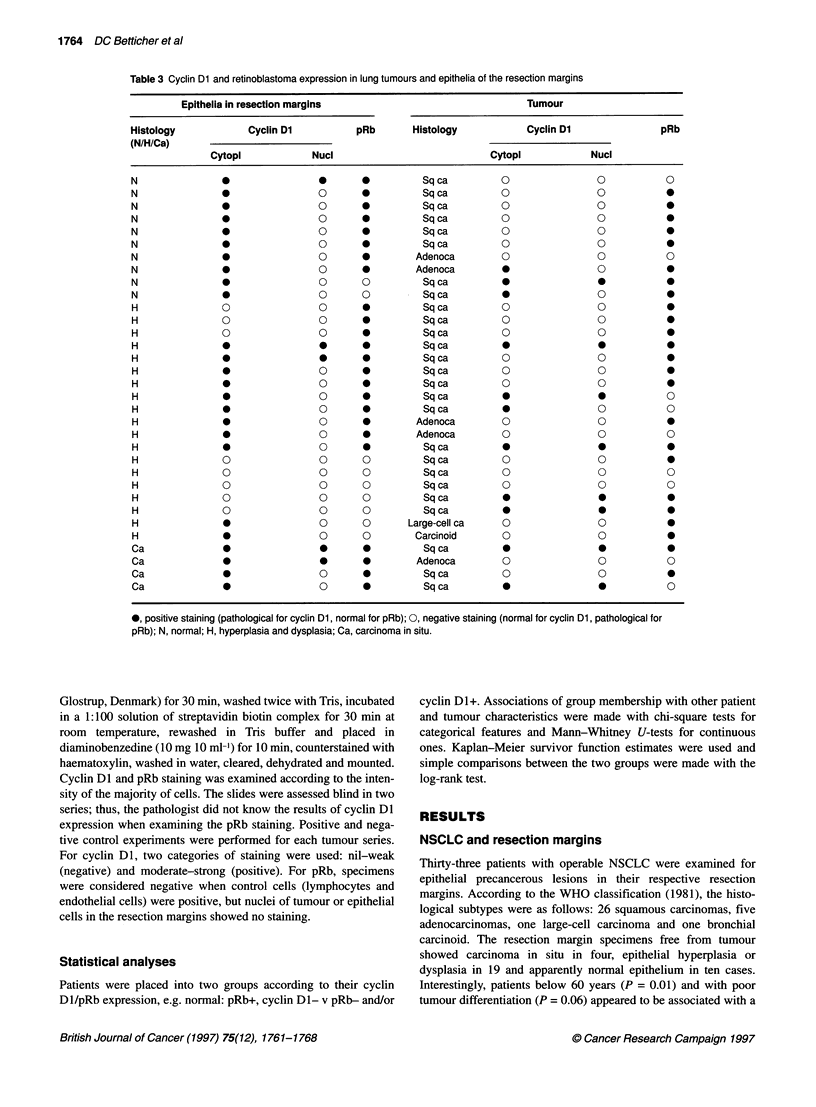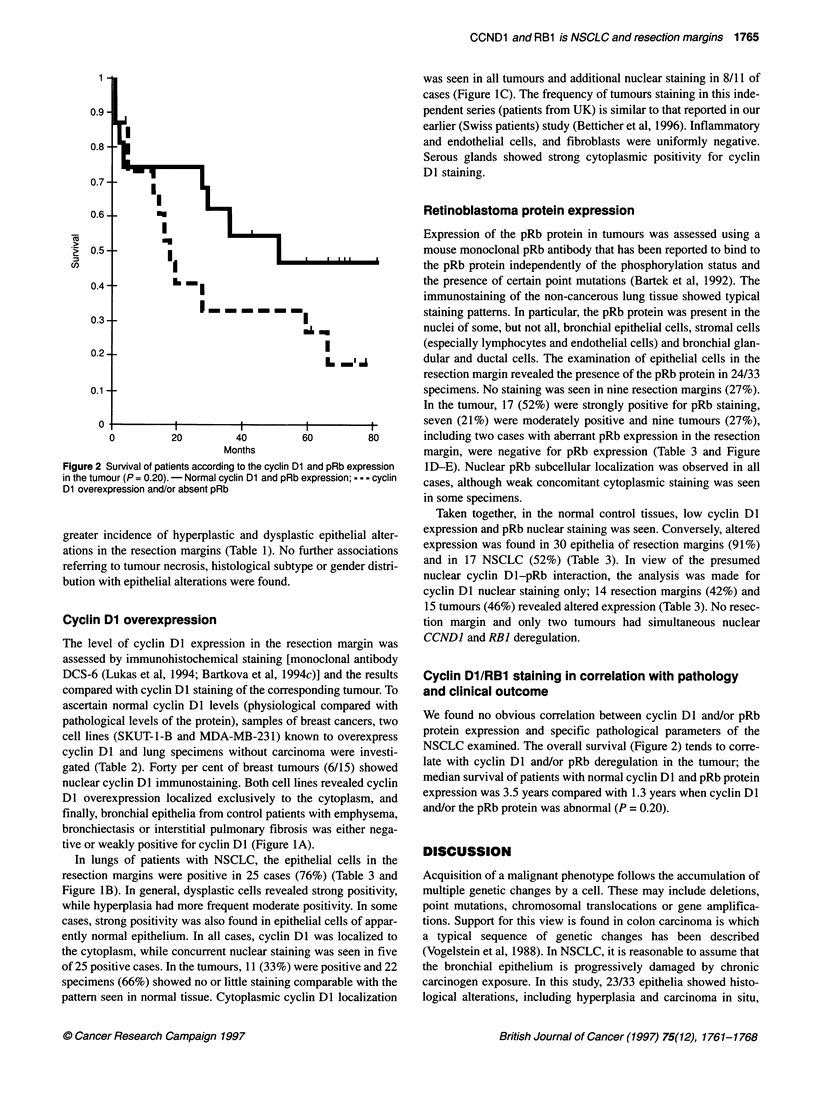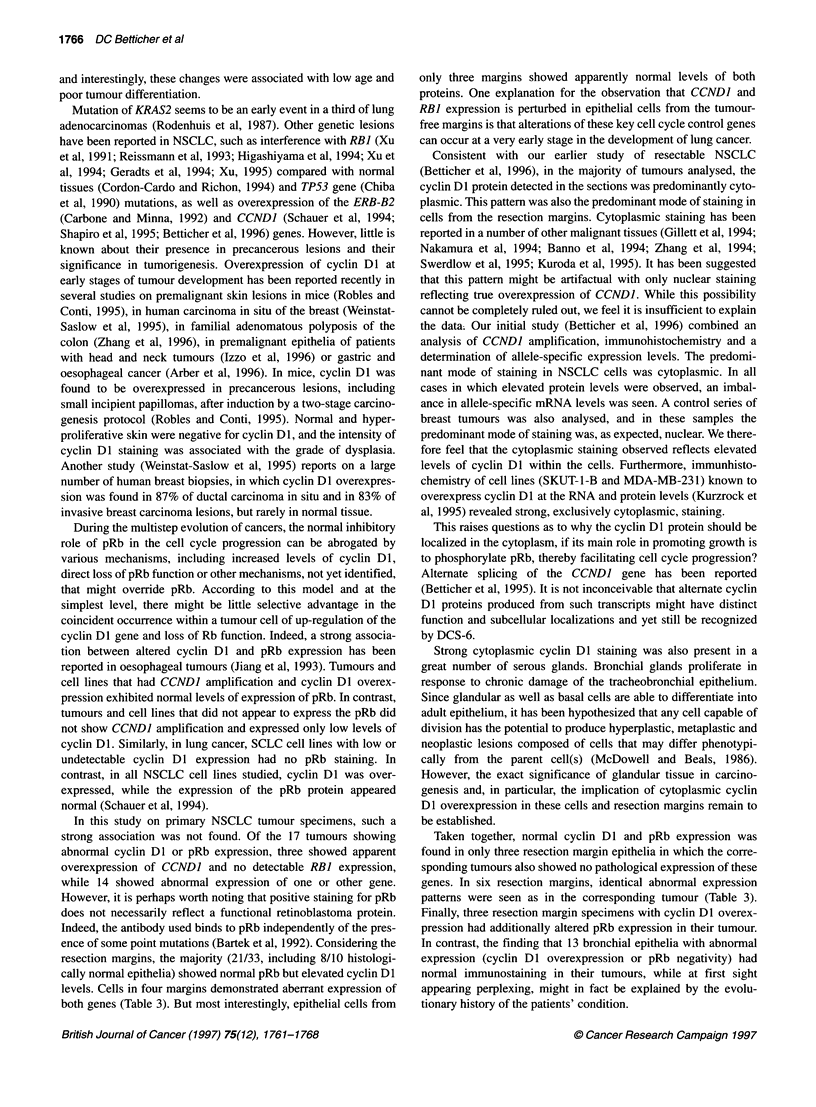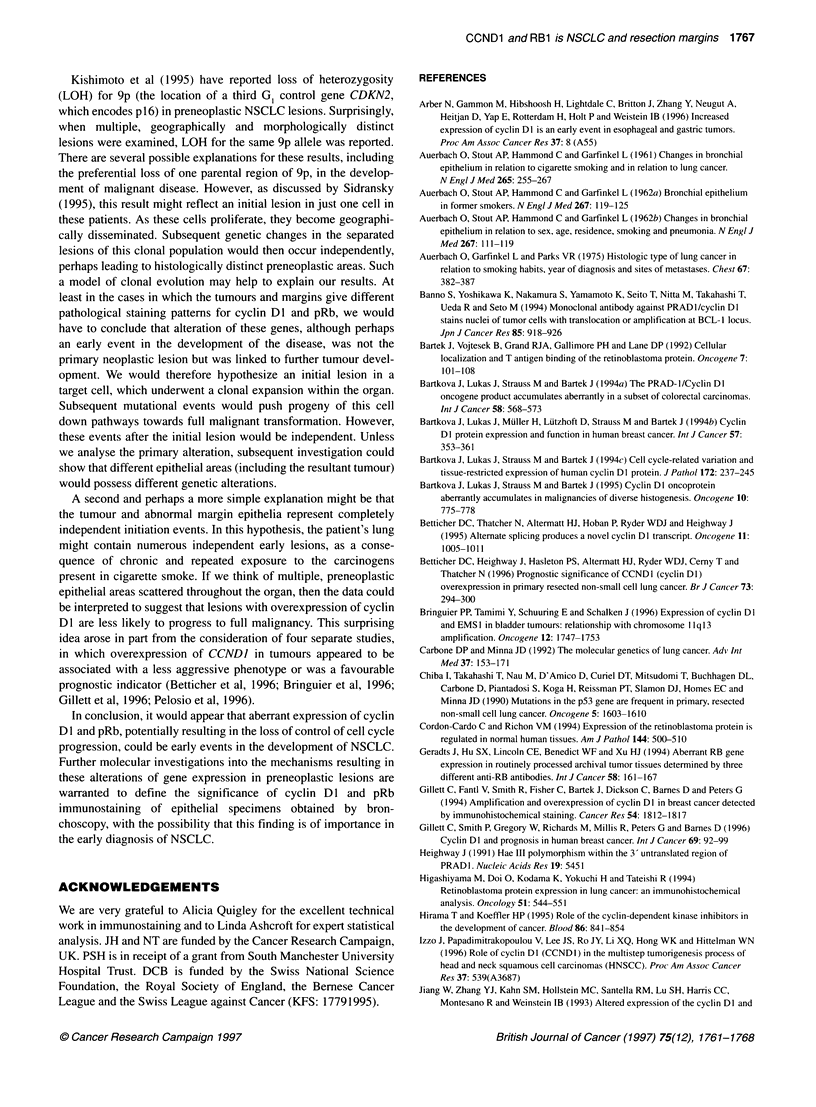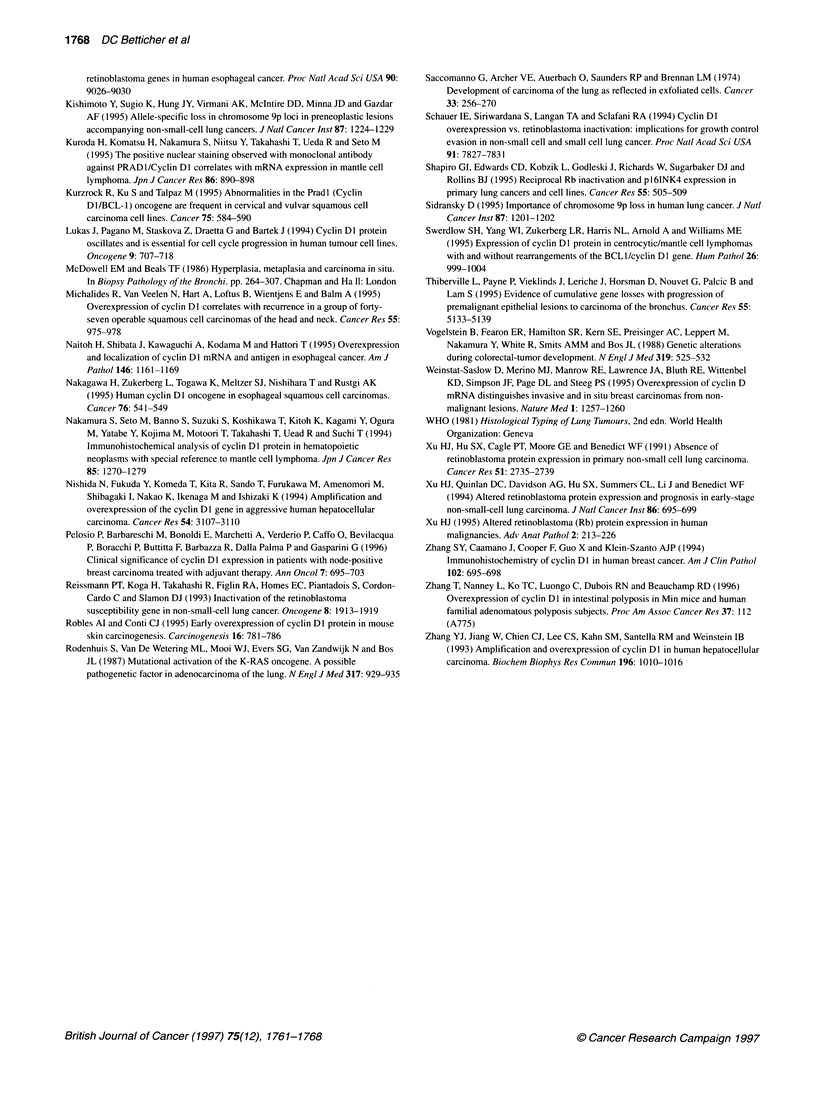# Abnormal expression of CCND1 and RB1 in resection margin epithelia of lung cancer patients.

**DOI:** 10.1038/bjc.1997.300

**Published:** 1997

**Authors:** D. C. Betticher, J. Heighway, N. Thatcher, P. S. Hasleton

**Affiliations:** CRC Department of Med. Oncology, Christie Hospital (NHS) Trust, Manchester, UK.

## Abstract

**Images:**


British Journal of Cancer (1997) 75(12), 1761–1768
© 1997 Cancer Research Campaign

### Abnormal expression of *CCND1* and *RB1* in resection margin epithelia of lung cancer patients

**DC Betticher[1,2], J Heighway[3], N Thatcher[1], PS Hasleton[4]**

[1]CRC Department of Med. Oncology, Christie Hospital (NHS) Trust, Wilmslow Road, Manchester, UK; [2]University Hospital of Berne, Switzerland; [3]CRC Department of Cancer Genetics, Paterson Institute for Cancer Research, Christie Hospital (NHS) Trust, Wilmslow Road, Manchester, UK; [4]Pathology Department, Wythenshawe Hospital, Manchester, UK

**Summary** Tumours develop through the accumulation of genetic alterations associated with a progressive increase of the malignant phenotype. In lung cancer, chronic exposure of bronchial epithelium to carcinogens in cigarette smoke may lead to multiple dysplastic and hyperplastic lesions scattered throughout the tracheobronchial tree. Little is known about the genetic alterations in such lesions. This study was carried out to examine cyclin D1 (*CCND1*) and retinoblastoma (*RB1*) gene expression in the bronchial epithelium of patients with lung cancer. Lung tumours and their corresponding tumour-free resection margins from 33 patients who underwent resection of non-small-cell lung cancer (NSCLC) were examined by immunostaining with monoclonal antibodies against cyclin D1 (DCS-6; Novocastra) and pRb (NCL Rb-1; Novocastra). Examination of the resection margins revealed four carcinomas in situ, 19 hyperplasias and ten sections showing apparently normal bronchial epithelium. A control group of patients, without lung tumours and who had never smoked, revealed no or weak cyclin D1 and positive pRb staining within bronchial epithelia. Increased cyclin D1 and diminished pRb expression were found in 76% ($n$ = 25) and 27% ($n$ = 9) of the resection margins respectively, and in 12% ($n$ = 4) both cyclin D1 and pRb expression were altered. In the corresponding tumours, 48% ($n$ = 16) were normal, while altered expression was found for cyclin D1 in 33% ($n$ = 11), pRb in 27% ($n$ = 9) and both in 9% ($n$ = 3) of cases. It appears that altered expression of cyclin D1 and pRb is an early event in NSCLC development in almost half of cases analysed. Further investigations are needed to determine the significance of immunostaining of bronchial specimens in individuals at risk of lung cancer, with the possibility that the observations are of importance in the early diagnosis of NSCLC.

**Keywords:** non-small-cell lung cancer; carcinogenesis; cyclin D1; *CCND1*; retinoblastoma protein; *RB1*; carcinoma in situ

Lung cancer has become a worldwide problem with a greater than tenfold increase in incidence of reported disease since 1930. Chronic exposure to bronchial irritants appears to lead to epithelial changes, scattered throughout the tracheobronchial tree (Auerbach et al, 1962*a,b*, 1975). Patients with lung cancer have a much greater frequency of epithelial hyperplasia in main bronchi (>90%) compared with patients (10%) who have never smoked (Auerbach et al, 1961). The best evidence for an association between carcinoma in situ and invasive carcinoma probably comes from sputum cytology from uranium miners, which showed increasingly abnormal epithelial cells as the patients progressed towards invasive lung tumours (Saccomanno et al, 1974). These results suggest that the whole tracheobronchial tree is affected by carcinogen exposure. Cells with genetic lesions resulting in a growth advantage are likely to replace the epithelium of the whole tracheobronchial tree and, in the case of additional genetic events, may show invasive growth (Thiberville et al, 1995).

Cyclins, through the targeting of cyclin-dependent kinases (CDKs), control progression of the cell during the various stages of the cell cycle. With respect to cancer, perhaps the most important of these proteins is cyclin D1. Cyclin D1–CDK4 complexes appear to act by phosphorylating and inactivating the retinoblastoma-suppressor protein (pRb). This results in the release, from pRb, of a bound transcription factor E2F. E2F complexes then activate genes necessary for cell division. The p16 protein suppresses the process by competitively binding to the CDK4 molecule. Component genes of this control pathway are frequently mutated, amplified or deleted in malignant cells (for review see Hirama and Koeffler, 1995). Overexpression of cyclin D1 has been reported in epithelial tumours, such as colorectal, head and neck, oesophageal, breast, uterus, hepatocellular and lung carcinomas, melanomas and sarcomas (Zhang et al, 1993; Bartkova et al, 1994 *a,b*, 1995; Gillett et al, 1994; Nishida et al, 1994; Michalides et al, 1995; Naitoh et al, 1995; Nakagawa et al, 1995). We have recently reported cyclin D1 overexpression in 43% of non-small-cell lung cancers (NSCLC) (Betticher et al, 1996). The overexpression was caused by *CCND1* amplification in only 17% of cases. However, in all cases showing overexpression and informative for a *Hae*III polymorphism (Heighway, 1991), an imbalance in allele-specific expression was observed. This suggested specific up-regulation of one *CCND1* allele and was consistent with the gene having a key function in lung carcinogenesis.

Cyclin D1 and pRb are part of a complicated network that governs cell proliferation. During the last years new proteins (p16, p15, p57, p27 and p21) were reported to possess inhibitory activity on the cyclin–kinase complexes (Hirama and Koeffler, 1995). However, since cyclin D1–CDK4–pRb stimulates the proliferation and function before the commitment point, such a deregulation might have primordial importance in malignant growth. We were, therefore,

*Received 6 August 1996*
*Revised 6 November 1996*
*Accepted 20 November 1996*

*Correspondence to:* N Thatcher, CRC Department of Medical Oncology, Christie Hospital, Wilmslow Road, Withington, Manchester M20 4BX, UK

**Table 1** Association of epithelial alterations in the resection margins of NSCLC and patient/tumour characteristics (chi-square test)

| | Altered epithelium (hyper-, dysplasia, carcinoma in situ) | Normal epithelium | P-value |
|---|---|---|---|
| Number of patients | 23 | 10 | |
| Sex | | | 0.75 |
| Male | 19 | 8 | |
| Female | 4 | 2 | |
| Age | | | 0.01 |
| < 60 years | 12 | 0 | |
| ≥ 60 years | 11 | 10 | |
| Histology | | | 0.73 |
| Squamous carcinoma | 18 | 8 | |
| Non-squamous carcinoma | 5 | 2 | |
| Differentiation | | | 0.06 |
| Good–moderate | 11 | 9 | |
| Poor | 12 | 1 | |
| Necrosis | | | 0.97 |
| Marked | 10 | 5 | |
| Scant | 13 | 5 | |
| Lymphocytic infiltration of the tumour | | | 0.50 |
| Prominent | 3 | 3 | |
| Moderate–poor | 20 | 7 | |

**Table 2** Cyclin D1 immunostaining in breast cancer, *normal lung epithelium from patients who underwent lung transplantation for emphysema, fibrosis and bronchiectasis, and from NSCLC with their respective resection margins

| Tissue/tumour/cell line | Number of samples examined | Cyclin D1 immunostaining pattern | | | | |
|---|---|---|---|---|---|---|
| | | Nil | Weak | Mod–strong | | |
| | | | | N | C | N+C |
| Breast cancer | 15 | 9 | – | 6 | – | – |
| Normal epithelium* | 6 | 3 | 3 | – | – | – |
| Cell lines | | | | | | |
| SKUT-1-B (leiomyosarcoma) | | – | – | – | + | – |
| MDA-MB-231 (breast cancer) | | – | – | – | + | – |
| NSCLC | | | | | | |
| Tumour | 33 | 6 | 16 | – | 3 | 8 |
| Resection margin | 33 | 1 | 7 | – | 20 | 5 |

N, nuclear; C, cytoplasmic; N+C, both.

interested to study whether an alteration of *CCND1* and *RB1* gene expression would occur early in lung tumour development.

### PATIENTS AND METHODS

### Patient characteristics and specimens

Tumour and resection margin samples were obtained from 33 consecutive patients [27 men, six women, median age 63 years (range 39–77 years)] who underwent resection of NSCLC at the Regional Cardiothoracic Centre, Wythenshawe Hospital, Manchester, UK. They had received no chemo- or radiotherapy before surgery. All tumours were classified according to the standard WHO criteria (1981). The degree of lymphocytic infiltration, presence of necrosis and vascular infiltration were determined histologically. Eleven of 31 patients with survival data are alive with a median follow-up of 70 months (range 3–77 months).

The specificity of immunostaining was determined for 14 breast cancer specimens as reported previously (Betticher et al, 1996), in two cells lines (SKUT-1-B and MDA-MB-231) known to over-express cyclin D1, and in six lung specimens from patients with no lung tumours and who had never smoked. In these patients, lung transplantation was performed because of emphysema ($n$ = 3), interstitial pulmonary fibrosis ($n$ = 2) and bronchiectasis ($n$ = 1).

### Immunohistochemistry

The immunohistochemistry was performed as described previously (Gillett et al, 1994; Geradts et al, 1994; Betticher et al, 1996). Briefly, 4-μm formalin-fixed paraffin sections from tumour

 © Cancer Research Campaign 1997

**Figure 1** (**A–C**) Cyclin D1 expression in (**A**) normal epithelium of lung without tumour (cyclin D1–); (**B**) hyperplastic epithelium of a lung with NSCLC (cytoplasmic cyclin D1 overexpression); (**C**) squamous lung tumour (cyclin D1 nuclear and cytoplasmic overexpression); (**D–E**) retinoblastoma expression in (**D**) carcinoma in situ (pRb+) and (**E**) lung tumour (pRb–)

(breast cancer, resection margins with respective NSCLC) and lung specimens were air dried on 2% APTS (Sigma, Poole, UK) coated slides. After dewaxing in xylene, the sections were treated for 15 min with 300 ml of methanol and 10 ml of hydrogen peroxide to block endogenous peroxidase and rinsed thoroughly in water. They were then placed in citrate buffer, boiled twice in a microwave, washed with water and placed in Tris buffer (pH 7.6). After incubation in 1:100 goat serum for 20 min at room temperature, they were placed either in 1% bovine serum albumin (BSA) and 1:100 mouse monoclonal cyclin D1 antibody (DCS-6; Novocastra, Newcastle, UK) or in 1% BSA and 1:50 mouse monoclonal pRb antibody (NCL-Rb1; Novocastra) overnight at room temperature. After two washes with Tris buffer, they were incubated in 1:100 biotinylated goat anti-mouse/rabbit IG (Dako,

**Table 3** Cyclin D1 and retinoblastoma expression in lung tumours and epithelia of the resection margins

| Epithelia in resection margins | | | | Tumour | | | |
|---|---|---|---|---|---|---|---|
| Histology (N/H/Ca) | Cyclin D1 | | pRb | Histology | Cyclin D1 | | pRb |
| | Cytopl | Nucl | | | Cytopl | Nucl | |
| N | ● | ● | ● | Sq ca | ○ | ○ | ○ |
| N | ● | ○ | ● | Sq ca | ○ | ○ | ● |
| N | ● | ○ | ● | Sq ca | ○ | ○ | ● |
| N | ● | ○ | ● | Sq ca | ○ | ○ | ● |
| N | ● | ○ | ● | Sq ca | ○ | ○ | ● |
| N | ● | ○ | ● | Sq ca | ○ | ○ | ● |
| N | ● | ○ | ● | Adenoca | ○ | ○ | ○ |
| N | ● | ○ | ● | Adenoca | ● | ○ | ● |
| N | ● | ○ | ○ | Sq ca | ● | ● | ● |
| N | ● | ○ | ○ | Sq ca | ● | ○ | ● |
| H | ○ | ○ | ● | Sq ca | ○ | ○ | ● |
| H | ○ | ○ | ● | Sq ca | ○ | ○ | ● |
| H | ○ | ○ | ● | Sq ca | ○ | ○ | ● |
| H | ● | ● | ● | Sq ca | ● | ● | ● |
| H | ● | ● | ● | Sq ca | ○ | ○ | ● |
| H | ● | ○ | ● | Sq ca | ○ | ○ | ● |
| H | ● | ○ | ● | Sq ca | ○ | ○ | ● |
| H | ● | ○ | ● | Sq ca | ● | ● | ○ |
| H | ● | ○ | ● | Sq ca | ● | ○ | ○ |
| H | ● | ○ | ● | Adenoca | ○ | ○ | ● |
| H | ● | ○ | ● | Adenoca | ○ | ○ | ○ |
| H | ● | ○ | ● | Sq ca | ● | ● | ● |
| H | ○ | ○ | ○ | Sq ca | ○ | ○ | ● |
| H | ○ | ○ | ○ | Sq ca | ○ | ○ | ○ |
| H | ○ | ○ | ○ | Sq ca | ○ | ○ | ○ |
| H | ○ | ○ | ○ | Sq ca | ● | ● | ● |
| H | ○ | ○ | ○ | Sq ca | ● | ● | ● |
| H | ● | ○ | ○ | Large-cell ca | ○ | ○ | ● |
| H | ● | ○ | ○ | Carcinoid | ○ | ○ | ● |
| Ca | ● | ● | ● | Sq ca | ● | ● | ● |
| Ca | ● | ● | ● | Adenoca | ○ | ○ | ○ |
| Ca | ● | ○ | ● | Sq ca | ○ | ○ | ● |
| Ca | ● | ○ | ● | Sq ca | ● | ● | ○ |

●, positive staining (pathological for cyclin D1, normal for pRb); ○, negative staining (normal for cyclin D1, pathological for pRb); N, normal; H, hyperplasia and dysplasia; Ca, carcinoma in situ.

Glostrup, Denmark) for 30 min, washed twice with Tris, incubated in a 1:100 solution of streptavidin biotin complex for 30 min at room temperature, rewashed in Tris buffer and placed in diaminobenzedine (10 mg 10 $ml^{-1}$) for 10 min, counterstained with haematoxylin, washed in water, cleared, dehydrated and mounted. Cyclin D1 and pRb staining was examined according to the intensity of the majority of cells. The slides were assessed blind in two series; thus, the pathologist did not know the results of cyclin D1 expression when examining the pRb staining. Positive and negative control experiments were performed for each tumour series. For cyclin D1, two categories of staining were used: nil–weak (negative) and moderate–strong (positive). For pRb, specimens were considered negative when control cells (lymphocytes and endothelial cells) were positive, but nuclei of tumour or epithelial cells in the resection margins showed no staining.

### Statistical analyses

Patients were placed into two groups according to their cyclin D1/pRb expression, e.g. normal: pRb+, cyclin D1– v pRb– and/or cyclin D1+. Associations of group membership with other patient and tumour characteristics were made with chi-square tests for categorical features and Mann–Whitney *U*-tests for continuous ones. Kaplan–Meier survivor function estimates were used and simple comparisons between the two groups were made with the log-rank test.

### RESULTS

### NSCLC and resection margins

Thirty-three patients with operable NSCLC were examined for epithelial precancerous lesions in their respective resection margins. According to the WHO classification (1981), the histological subtypes were as follows: 26 squamous carcinomas, five adenocarcinomas, one large-cell carcinoma and one bronchial carcinoid. The resection margin specimens free from tumour showed carcinoma in situ in four, epithelial hyperplasia or dysplasia in 19 and apparently normal epithelium in ten cases. Interestingly, patients below 60 years ($P = 0.01$) and with poor tumour differentiation ($P = 0.06$) appeared to be associated with a

*© Cancer Research Campaign 1997*

**Figure 2** Survival of patients according to the cyclin D1 and pRb expression in the tumour ($P = 0.20$). — Normal cyclin D1 and pRb expression; ▪▪▪ cyclin D1 overexpression and/or absent pRb greater incidence of hyperplastic and dysplastic epithelial alterations in the resection margins (Table 1). No further associations referring to tumour necrosis, histological subtype or gender distribution with epithelial alterations were found.

### Cyclin D1 overexpression

The level of cyclin D1 expression in the resection margin was assessed by immunohistochemical staining [monoclonal antibody DCS-6 (Lukas et al, 1994; Bartkova et al, 1994*c*)] and the results compared with cyclin D1 staining of the corresponding tumour. To ascertain normal cyclin D1 levels (physiological compared with pathological levels of the protein), samples of breast cancers, two cell lines (SKUT-1-B and MDA-MB-231) known to overexpress cyclin D1 and lung specimens without carcinoma were investigated (Table 2). Forty per cent of breast tumours (6/15) showed nuclear cyclin D1 immunostaining. Both cell lines revealed cyclin D1 overexpression localized exclusively to the cytoplasm, and finally, bronchial epithelia from control patients with emphysema, bronchiectasis or interstitial pulmonary fibrosis was either negative or weakly positive for cyclin D1 (Figure 1A).

In lungs of patients with NSCLC, the epithelial cells in the resection margins were positive in 25 cases (76%) (Table 3 and Figure 1B). In general, dysplastic cells revealed strong positivity, while hyperplasia had more frequent moderate positivity. In some cases, strong positivity was also found in epithelial cells of apparently normal epithelium. In all cases, cyclin D1 was localized to the cytoplasm, while concurrent nuclear staining was seen in five of 25 positive cases. In the tumours, 11 (33%) were positive and 22 specimens (66%) showed no or little staining comparable with the pattern seen in normal tissue. Cytoplasmic cyclin D1 localization was seen in all tumours and additional nuclear staining in 8/11 of cases (Figure 1C). The frequency of tumours staining in this independent series (patients from UK) is similar to that reported in our earlier (Swiss patients) study (Betticher et al, 1996). Inflammatory and endothelial cells, and fibroblasts were uniformly negative. Serous glands showed strong cytoplasmic positivity for cyclin D1 staining.

### Retinoblastoma protein expression

Expression of the pRb protein in tumours was assessed using a mouse monoclonal pRb antibody that has been reported to bind to the pRb protein independently of the phosphorylation status and the presence of certain point mutations (Bartek et al, 1992). The immunostaining of the non-cancerous lung tissue showed typical staining patterns. In particular, the pRb protein was present in the nuclei of some, but not all, bronchial epithelial cells, stromal cells (especially lymphocytes and endothelial cells) and bronchial glandular and ductal cells. The examination of epithelial cells in the resection margin revealed the presence of the pRb protein in 24/33 specimens. No staining was seen in nine resection margins (27%). In the tumour, 17 (52%) were strongly positive for pRb staining, seven (21%) were moderately positive and nine tumours (27%), including two cases with aberrant pRb expression in the resection margin, were negative for pRb expression (Table 3 and Figure 1D–E). Nuclear pRb subcellular localization was observed in all cases, although weak concomitant cytoplasmic staining was seen in some specimens.

Taken together, in the normal control tissues, low cyclin D1 expression and pRb nuclear staining was seen. Conversely, altered expression was found in 30 epithelia of resection margins (91%) and in 17 NSCLC (52%) (Table 3). In view of the presumed nuclear cyclin D1–pRb interaction, the analysis was made for cyclin D1 nuclear staining only; 14 resection margins (42%) and 15 tumours (46%) revealed altered expression (Table 3). No resection margin and only two tumours had simultaneous nuclear *CCND1* and *RB1* deregulation.

### Cyclin D1/RB1 staining in correlation with pathology and clinical outcome

We found no obvious correlation between cyclin D1 and/or pRb protein expression and specific pathological parameters of the NSCLC examined. The overall survival (Figure 2) tends to correlate with cyclin D1 and/or pRb deregulation in the tumour; the median survival of patients with normal cyclin D1 and pRb protein expression was 3.5 years compared with 1.3 years when cyclin D1 and/or the pRb protein was abnormal ($P = 0.20$).

### DISCUSSION

Acquisition of a malignant phenotype follows the accumulation of multiple genetic changes by a cell. These may include deletions, point mutations, chromosomal translocations or gene amplifications. Support for this view is found in colon carcinoma is which a typical sequence of genetic changes has been described (Vogelstein et al, 1988). In NSCLC, it is reasonable to assume that the bronchial epithelium is progressively damaged by chronic carcinogen exposure. In this study, 23/33 epithelia showed histological alterations, including hyperplasia and carcinoma in situ,

© Cancer Research Campaign 1997 

and interestingly, these changes were associated with low age and poor tumour differentiation.

Mutation of *KRAS2* seems to be an early event in a third of lung adenocarcinomas (Rodenhuis et al, 1987). Other genetic lesions have been reported in NSCLC, such as interference with *RB1* (Xu et al, 1991; Reissmann et al, 1993; Higashiyama et al, 1994; Xu et al, 1994; Geradts et al, 1994; Xu, 1995) compared with normal tissues (Cordon-Cardo and Richon, 1994) and *TP53* gene (Chiba et al, 1990) mutations, as well as overexpression of the *ERB-B2* (Carbone and Minna, 1992) and *CCND1* (Schauer et al, 1994; Shapiro et al, 1995; Betticher et al, 1996) genes. However, little is known about their presence in precancerous lesions and their significance in tumorigenesis. Overexpression of cyclin D1 at early stages of tumour development has been reported recently in several studies on premalignant skin lesions in mice (Robles and Conti, 1995), in human carcinoma in situ of the breast (Weinstat-Saslow et al, 1995), in familial adenomatous polyposis of the colon (Zhang et al, 1996), in premalignant epithelia of patients with head and neck tumours (Izzo et al, 1996) or gastric and oesophageal cancer (Arber et al, 1996). In mice, cyclin D1 was found to be overexpressed in precancerous lesions, including small incipient papillomas, after induction by a two-stage carcinogenesis protocol (Robles and Conti, 1995). Normal and hyperproliferative skin were negative for cyclin D1, and the intensity of cyclin D1 staining was associated with the grade of dysplasia. Another study (Weinstat-Saslow et al, 1995) reports on a large number of human breast biopsies, in which cyclin D1 overexpression was found in 87% of ductal carcinoma in situ and in 83% of invasive breast carcinoma lesions, but rarely in normal tissue.

During the multistep evolution of cancers, the normal inhibitory role of pRb in the cell cycle progression can be abrogated by various mechanisms, including increased levels of cyclin D1, direct loss of pRb function or other mechanisms, not yet identified, that might override pRb. According to this model and at the simplest level, there might be little selective advantage in the coincident occurrence within a tumour cell of up-regulation of the cyclin D1 gene and loss of Rb function. Indeed, a strong association between altered cyclin D1 and pRb expression has been reported in oesophageal tumours (Jiang et al, 1993). Tumours and cell lines that had *CCND1* amplification and cyclin D1 overexpression exhibited normal levels of expression of pRb. In contrast, tumours and cell lines that did not appear to express the pRb did not show *CCND1* amplification and expressed only low levels of cyclin D1. Similarly, in lung cancer, SCLC cell lines with low or undetectable cyclin D1 expression had no pRb staining. In contrast, in all NSCLC cell lines studied, cyclin D1 was overexpressed, while the expression of the pRb protein appeared normal (Schauer et al, 1994).

In this study on primary NSCLC tumour specimens, such a strong association was not found. Of the 17 tumours showing abnormal cyclin D1 or pRb expression, three showed apparent overexpression of *CCND1* and no detectable *RB1* expression, while 14 showed abnormal expression of one or other gene. However, it is perhaps worth noting that positive staining for pRb does not necessarily reflect a functional retinoblastoma protein. Indeed, the antibody used binds to pRb independently of the presence of some point mutations (Bartek et al, 1992). Considering the resection margins, the majority (21/33, including 8/10 histologically normal epithelia) showed normal pRb but elevated cyclin D1 levels. Cells in four margins demonstrated aberrant expression of both genes (Table 3). But most interestingly, epithelial cells from only three margins showed apparently normal levels of both proteins. One explanation for the observation that *CCND1* and *RB1* expression is perturbed in epithelial cells from the tumour-free margins is that alterations of these key cell cycle control genes can occur at a very early stage in the development of lung cancer.

Consistent with our earlier study of resectable NSCLC (Betticher et al, 1996), in the majority of tumours analysed, the cyclin D1 protein detected in the sections was predominantly cytoplasmic. This pattern was also the predominant mode of staining in cells from the resection margins. Cytoplasmic staining has been reported in a number of other malignant tissues (Gillett et al, 1994; Nakamura et al, 1994; Banno et al, 1994; Zhang et al, 1994; Swerdlow et al, 1995; Kuroda et al, 1995). It has been suggested that this pattern might be artifactual with only nuclear staining reflecting true overexpression of *CCND1*. While this possibility cannot be completely ruled out, we feel it is insufficient to explain the data. Our initial study (Betticher et al, 1996) combined an analysis of *CCND1* amplification, immunohistochemistry and a determination of allele-specific expression levels. The predominant mode of staining in NSCLC cells was cytoplasmic. In all cases in which elevated protein levels were observed, an imbalance in allele-specific mRNA levels was seen. A control series of breast tumours was also analysed, and in these samples the predominant mode of staining was, as expected, nuclear. We therefore feel that the cytoplasmic staining observed reflects elevated levels of cyclin D1 within the cells. Furthermore, immunhistochemistry of cell lines (SKUT-1-B and MDA-MB-231) known to overexpress cyclin D1 at the RNA and protein levels (Kurzrock et al, 1995) revealed strong, exclusively cytoplasmic, staining.

This raises questions as to why the cyclin D1 protein should be localized in the cytoplasm, if its main role in promoting growth is to phosphorylate pRb, thereby facilitating cell cycle progression? Alternate splicing of the *CCND1* gene has been reported (Betticher et al, 1995). It is not inconceivable that alternate cyclin D1 proteins produced from such transcripts might have distinct function and subcellular localizations and yet still be recognized by DCS-6.

Strong cytoplasmic cyclin D1 staining was also present in a great number of serous glands. Bronchial glands proliferate in response to chronic damage of the tracheobronchial epithelium. Since glandular as well as basal cells are able to differentiate into adult epithelium, it has been hypothesized that any cell capable of division has the potential to produce hyperplastic, metaplastic and neoplastic lesions composed of cells that may differ phenotypically from the parent cell(s) (McDowell and Beals, 1986). However, the exact significance of glandular tissue in carcinogenesis and, in particular, the implication of cytoplasmic cyclin D1 overexpression in these cells and resection margins remain to be established.

Taken together, normal cyclin D1 and pRb expression was found in only three resection margin epithelia in which the corresponding tumours also showed no pathological expression of these genes. In six resection margins, identical abnormal expression patterns were seen as in the corresponding tumour (Table 3). Finally, three resection margin specimens with cyclin D1 overexpression had additionally altered pRb expression in their tumour. In contrast, the finding that 13 bronchial epithelia with abnormal expression (cyclin D1 overexpression or pRb negativity) had normal immunostaining in their tumours, while at first sight appearing perplexing, might in fact be explained by the evolutionary history of the patients' condition.

© Cancer Research Campaign 1997

Kishimoto et al (1995) have reported loss of heterozygosity (LOH) for 9p (the location of a third $G_1$ control gene *CDKN2*, which encodes p16) in preneoplastic NSCLC lesions. Surprisingly, when multiple, geographically and morphologically distinct lesions were examined, LOH for the same 9p allele was reported. There are several possible explanations for these results, including the preferential loss of one parental region of 9p, in the development of malignant disease. However, as discussed by Sidransky (1995), this result might reflect an initial lesion in just one cell in these patients. As these cells proliferate, they become geographically disseminated. Subsequent genetic changes in the separated lesions of this clonal population would then occur independently, perhaps leading to histologically distinct preneoplastic areas. Such a model of clonal evolution may help to explain our results. At least in the cases in which the tumours and margins give different pathological staining patterns for cyclin D1 and pRb, we would have to conclude that alteration of these genes, although perhaps an early event in the development of the disease, was not the primary neoplastic lesion but was linked to further tumour development. We would therefore hypothesize an initial lesion in a target cell, which underwent a clonal expansion within the organ. Subsequent mutational events would push progeny of this cell down pathways towards full malignant transformation. However, these events after the initial lesion would be independent. Unless we analyse the primary alteration, subsequent investigation could show that different epithelial areas (including the resultant tumour) would possess different genetic alterations.

A second and perhaps a more simple explanation might be that the tumour and abnormal margin epithelia represent completely independent initiation events. In this hypothesis, the patient's lung might contain numerous independent early lesions, as a consequence of chronic and repeated exposure to the carcinogens present in cigarette smoke. If we think of multiple, preneoplastic epithelial areas scattered throughout the organ, then the data could be interpreted to suggest that lesions with overexpression of cyclin D1 are less likely to progress to full malignancy. This surprising idea arose in part from the consideration of four separate studies, in which overexpression of *CCND1* in tumours appeared to be associated with a less aggressive phenotype or was a favourable prognostic indicator (Betticher et al, 1996; Bringuier et al, 1996; Gillett et al, 1996; Pelosio et al, 1996).

In conclusion, it would appear that aberrant expression of cyclin D1 and pRb, potentially resulting in the loss of control of cell cycle progression, could be early events in the development of NSCLC. Further molecular investigations into the mechanisms resulting in these alterations of gene expression in preneoplastic lesions are warranted to define the significance of cyclin D1 and pRb immunostaining of epithelial specimens obtained by bronchoscopy, with the possibility that this finding is of importance in the early diagnosis of NSCLC.

### ACKNOWLEDGEMENTS

We are very grateful to Alicia Quigley for the excellent technical work in immunostaining and to Linda Ashcroft for expert statistical analysis. JH and NT are funded by the Cancer Research Campaign, UK. PSH is in receipt of a grant from South Manchester University Hospital Trust. DCB is funded by the Swiss National Science Foundation, the Royal Society of England, the Bernese Cancer League and the Swiss League against Cancer (KFS: 17791995).

### REFERENCES

Arber N, Gammon M, Hibshoosh H, Lightdale C, Britton J, Zhang Y, Neugut A, Heitjan D, Yap E, Rotterdam H, Holt P and Weistein IB (1996) Increased expression of cyclin D1 is an early event in esophageal and gastric tumors. *Proc Am Assoc Cancer Res* **37**: 8 (A55)

Auerbach O, Stout AP, Hammond C and Garfinkel L (1961) Changes in bronchial epithelium in relation to cigarette smoking and in relation to lung cancer. *N Engl J Med* **265**: 255–267

Auerbach O, Stout AP, Hammond C and Garfinkel L (1962*a*) Bronchial epithelium in former smokers. *N Engl J Med* **267**: 119–125

Auerbach O, Stout AP, Hammond C and Garfinkel L (1962*b*) Changes in bronchial epithelium in relation to sex, age, residence, smoking and pneumonia. *N Engl J Med* **267**: 111–119

Auerbach O, Garfinkel L and Parks VR (1975) Histologic type of lung cancer in relation to smoking habits, year of diagnosis and sites of metastases. *Chest* **67**: 382–387

Banno S, Yoshikawa K, Nakamura S, Yamamoto K, Seito T, Nitta M, Takahashi T, Ueda R and Seto M (1994) Monoclonal antibody against PRAD1/cyclin D1 stains nuclei of tumor cells with translocation or amplification at BCL-1 locus. *Jpn J Cancer Res* **85**: 918–926

Bartek J, Vojtesek B, Grand RJA, Gallimore PH and Lane DP (1992) Cellular localization and T antigen binding of the retinoblastoma protein. *Oncogene* **7**: 101–108

Bartkova J, Lukas J, Strauss M and Bartek J (1994*a*) The PRAD-1/Cyclin D1 oncogene product accumulates aberrantly in a subset of colorectal carcinomas. *Int J Cancer* **58**: 568–573

Bartkova J, Lukas J, Müller H, Lützhoft D, Strauss M and Bartek J (1994*b*) Cyclin D1 protein expression and function in human breast cancer. *Int J Cancer* **57**: 353–361

Bartkova J, Lukas J, Strauss M and Bartek J (1994*c*) Cell cycle-related variation and tissue-restricted expression of human cyclin D1 protein. *J Pathol* **172**: 237–245

Bartkova J, Lukas J, Strauss M and Bartek J (1995) Cyclin D1 oncoprotein aberrantly accumulates in malignancies of diverse histogenesis. *Oncogene* **10**: 775–778

Betticher DC, Thatcher N, Altermatt HJ, Hoban P, Ryder WDJ and Heighway J (1995) Alternate splicing produces a novel cyclin D1 transcript. *Oncogene* **11**: 1005–1011

Betticher DC, Heighway J, Hasleton PS, Altermatt HJ, Ryder WDJ, Cerny T and Thatcher N (1996) Prognostic significance of CCND1 (cyclin D1) overexpression in primary resected non-small cell lung cancer. *Br J Cancer* **73**: 294–300

Bringuier PP, Tamimi Y, Schuuring E and Schalken J (1996) Expression of cyclin D1 and EMS1 in bladder tumours: relationship with chromosome 11q13 amplification. *Oncogene* **12**: 1747–1753

Carbone DP and Minna JD (1992) The molecular genetics of lung cancer. *Adv Int Med* **37**: 153–171

Chiba I, Takahashi T, Nau M, D'Amico D, Curiel DT, Mitsudomi T, Buchhagen DL, Carbone D, Piantadosi S, Koga H, Reissman PT, Slamon DJ, Homes EC and Minna JD (1990) Mutations in the p53 gene are frequent in primary, resected non-small cell lung cancer. *Oncogene* **5**: 1603–1610

Cordon-Cardo C and Richon VM (1994) Expression of the retinoblastoma protein is regulated in normal human tissues. *Am J Pathol* **144**: 500–510

Geradts J, Hu SX, Lincoln CE, Benedict WF and Xu HJ (1994) Aberrant RB gene expression in routinely processed archival tumor tissues determined by three different anti-RB antibodies. *Int J Cancer* **58**: 161–167

Gillett C, Fantl V, Smith R, Fisher C, Bartek J, Dickson C, Barnes D and Peters G (1994) Amplification and overexpression of cyclin D1 in breast cancer detected by immunohistochemical staining. *Cancer Res* **54**: 1812–1817

Gillett C, Smith P, Gregory W, Richards M, Millis R, Peters G and Barnes D (1996) Cyclin D1 and prognosis in human breast cancer. *Int J Cancer* **69**: 92–99

Heighway J (1991) Hae III polymorphism within the 3′ untranslated region of PRAD1. *Nucleic Acids Res* **19**: 5451

Higashiyama M, Doi O, Kodama K, Yokuchi H and Tateishi R (1994) Retinoblastoma protein expression in lung cancer: an immunohistochemical analysis. *Oncology* **51**: 544–551

Hirama T and Koeffler HP (1995) Role of the cyclin-dependent kinase inhibitors in the development of cancer. *Blood* **86**: 841–854

Izzo J, Papadimitrakopoulou V, Lee JS, Ro JY, Li XQ, Hong WK and Hittelman WN (1996) Role of cyclin D1 (CCND1) in the multistep tumorigenesis process of head and neck squamous cell carcinomas (HNSCC). *Proc Am Assoc Cancer Res* **37**: 539(A3687)

Jiang W, Zhang YJ, Kahn SM, Hollstein MC, Santella RM, Lu SH, Harris CC, Montesano R and Weinstein IB (1993) Altered expression of the cyclin D1 and

© Cancer Research Campaign 1997

retinoblastoma genes in human esophageal cancer. *Proc Natl Acad Sci USA* **90**: 9026–9030

Kishimoto Y, Sugio K, Hung JY, Virmani AK, McIntire DD, Minna JD and Gazdar AF (1995) Allele-specific loss in chromosome 9p loci in preneoplastic lesions accompanying non-small-cell lung cancers. *J Natl Cancer Inst* **87**: 1224–1229

Kuroda H, Komatsu H, Nakamura S, Niitsu Y, Takahashi T, Ueda R and Seto M (1995) The positive nuclear staining observed with monoclonal antibody against PRAD1/Cyclin D1 correlates with mRNA expression in mantle cell lymphoma. *Jpn J Cancer Res* **86**: 890–898

Kurzrock R, Ku S and Talpaz M (1995) Abnormalities in the Prad1 (Cyclin D1/BCL-1) oncogene are frequent in cervical and vulvar squamous cell carcinoma cell lines. *Cancer* **75**: 584–590

Lukas J, Pagano M, Staskova Z, Draetta G and Bartek J (1994) Cyclin D1 protein oscillates and is essential for cell cycle progression in human tumour cell lines. *Oncogene* **9**: 707–718

McDowell EM and Beals TF (1986) Hyperplasia, metaplasia and carcinoma in situ. In *Biopsy Pathology of the Bronchi*. pp. 264–307. Chapman and Ha ll: London

Michalides R, Van Veelen N, Hart A, Loftus B, Wientjens E and Balm A (1995) Overexpression of cyclin D1 correlates with recurrence in a group of forty-seven operable squamous cell carcinomas of the head and neck. *Cancer Res* **55**: 975–978

Naitoh H, Shibata J, Kawaguchi A, Kodama M and Hattori T (1995) Overexpression and localization of cyclin D1 mRNA and antigen in esophageal cancer. *Am J Pathol* **146**: 1161–1169

Nakagawa H, Zukerberg L, Togawa K, Meltzer SJ, Nishihara T and Rustgi AK (1995) Human cyclin D1 oncogene in esophageal squamous cell carcinomas. *Cancer* **76**: 541–549

Nakamura S, Seto M, Banno S, Suzuki S, Koshikawa T, Kitoh K, Kagami Y, Ogura M, Yatabe Y, Kojima M, Motoori T, Takahashi T, Uead R and Suchi T (1994) Immunohistochemical analysis of cyclin D1 protein in hematopoietic neoplasms with special reference to mantle cell lymphoma. *Jpn J Cancer Res* **85**: 1270–1279

Nishida N, Fukuda Y, Komeda T, Kita R, Sando T, Furukawa M, Amenomori M, Shibagaki I, Nakao K, Ikenaga M and Ishizaki K (1994) Amplification and overexpression of the cyclin D1 gene in aggressive human hepatocellular carcinoma. *Cancer Res* **54**: 3107–3110

Pelosio P, Barbareschi M, Bonoldi E, Marchetti A, Verderio P, Caffo O, Bevilacqua P, Boracchi P, Buttitta F, Barbazza R, Dalla Palma P and Gasparini G (1996) Clinical significance of cyclin D1 expression in patients with node-positive breast carcinoma treated with adjuvant therapy. *Ann Oncol* **7**: 695–703

Reissmann PT, Koga H, Takahashi R, Figlin RA, Homes EC, Piantadois S, Cordon-Cardo C and Slamon DJ (1993) Inactivation of the retinoblastoma susceptibility gene in non-small-cell lung cancer. *Oncogene* **8**: 1913–1919

Robles AI and Conti CJ (1995) Early overexpression of cyclin D1 protein in mouse skin carcinogenesis. *Carcinogenesis* **16**: 781–786

Rodenhuis S, Van De Wetering ML, Mooi WJ, Evers SG, Van Zandwijk N and Bos JL (1987) Mutational activation of the K-RAS oncogene. A possible pathogenetic factor in adenocarcinoma of the lung. *N Engl J Med* **317**: 929–935

Saccomanno G, Archer VE, Auerbach O, Saunders RP and Brennan LM (1974) Development of carcinoma of the lung as reflected in exfoliated cells. *Cancer* **33**: 256–270

Schauer IE, Siriwardana S, Langan TA and Sclafani RA (1994) Cyclin D1 overexpression vs. retinoblastoma inactivation: implications for growth control evasion in non-small cell and small cell lung cancer. *Proc Natl Acad Sci USA* **91**: 7827–7831

Shapiro GI, Edwards CD, Kobzik L, Godleski J, Richards W, Sugarbaker DJ and Rollins BJ (1995) Reciprocal Rb inactivation and p16INK4 expression in primary lung cancers and cell lines. *Cancer Res* **55**: 505–509

Sidransky D (1995) Importance of chromosome 9p loss in human lung cancer. *J Natl Cancer Inst* **87**: 1201–1202

Swerdlow SH, Yang WI, Zukerberg LR, Harris NL, Arnold A and Williams ME (1995) Expression of cyclin D1 protein in centrocytic/mantle cell lymphomas with and without rearrangements of the BCL1/cyclin D1 gene. *Hum Pathol* **26**: 999–1004

Thiberville L, Payne P, Vieklinds J, Leriche J, Horsman D, Nouvet G, Palcic B and Lam S (1995) Evidence of cumulative gene losses with progression of premalignant epithelial lesions to carcinoma of the bronchus. *Cancer Res* **55**: 5133–5139

Vogelstein B, Fearon ER, Hamilton SR, Kern SE, Preisinger AC, Leppert M, Nakamura Y, White R, Smits AMM and Bos JL (1988) Genetic alterations during colorectal-tumor development. *N Engl J Med* **319**: 525–532

Weinstat-Saslow D, Merino MJ, Manrow RE, Lawrence JA, Bluth RE, Wittenbel KD, Simpson JF, Page DL and Steeg PS (1995) Overexpression of cyclin D mRNA distinguishes invasive and in situ breast carcinomas from non-malignant lesions. *Nature Med* **1**: 1257–1260

WHO (1981) *Histological Typing of Lung Tumours*, 2nd edn. World Health Organization: Geneva

Xu HJ, Hu SX, Cagle PT, Moore GE and Benedict WF (1991) Absence of retinoblastoma protein expression in primary non-small cell lung carcinoma. *Cancer Res* **51**: 2735–2739

Xu HJ, Quinlan DC, Davidson AG, Hu SX, Summers CL, Li J and Benedict WF (1994) Altered retinoblastoma protein expression and prognosis in early-stage non-small-cell lung carcinoma. *J Natl Cancer Inst* **86**: 695–699

Xu HJ (1995) Altered retinoblastoma (Rb) protein expression in human malignancies. *Adv Anat Pathol* **2**: 213–226

Zhang SY, Caamano J, Cooper F, Guo X and Klein-Szanto AJP (1994) Immunohistochemistry of cyclin D1 in human breast cancer. *Am J Clin Pathol* **102**: 695–698

Zhang T, Nanney L, Ko TC, Luongo C, Dubois RN and Beauchamp RD (1996) Overexpression of cyclin D1 in intestinal polyposis in Min mice and human familial adenomatous polyposis subjects. *Proc Am Assoc Cancer Res* **37**: 112 (A775)

Zhang YJ, Jiang W, Chien CJ, Lee CS, Kahn SM, Santella RM and Weinstein IB (1993) Amplification and overexpression of cyclin D1 in human hepatocellular carcinoma. *Biochem Biophys Res Commun* **196**: 1010–1016

© Cancer Research Campaign 1997